# Online Ambassador Visits for Hospitalized Children With Cancer: Qualitative Evaluation of Implementation

**DOI:** 10.2196/53309

**Published:** 2024-09-04

**Authors:** Natasha Nybro Boensvang, Mette Weibel, Claire E Wakefield, Pernille Envold Bidstrup, Marianne Olsen, Karin Bækgaard Nissen, Vibeke Spager, Martin Kaj Fridh, Hanne Bækgaard Larsen

**Affiliations:** 1 Faculty of Health and Medical Sciences University of Copenhagen Copenhagen Denmark; 2 Department of Paediatrics and Adolescent Medicine University Hospital of Copehagen (Rigshospitalet) Copenhagen Denmark; 3 Behavioral Sciences Unit Kids Cancer Centre Sydney Children's Hospital Sydney Australia; 4 School for Women's and Children's Health Faculty of Medicine and Health University of New South Wales Sydney Australia; 5 Psychological Aspects of Cancer Danish Cancer Institute Danish Cancer Society Copenhagen Denmark; 6 Department of Psychology University of Copenhagen Copenhagen Denmark; 7 Department of Paediatrics and Adolescent Medicine Paediatric Haematology and Oncology Section University Hospital Aalborg Aalborg Denmark; 8 Department of Paediatric and Adolescent Medicine University Hospital Aarhus Aarhus Denmark; 9 Faculty of Health and Medical Sciences University of Copenhagen København Denmark; 10 The Paediatric Clinic Juliane Marie Centre University Hospital of Copenhagen Copenhagen Denmark

**Keywords:** Children, cancer, school-aged, peers, interaction, online, in-hospital, social, relationship, quality of life, intervention, qualiative

## Abstract

**Background:**

Children with cancer or cancer-like disease risk treatment-related isolation, which can negatively impact their peer relationships and social competencies and exacerbate their loneliness. During the COVID-19 pandemic, increased online socialization became the new normal imposed by national isolation guidelines. To adhere to the treatment-related isolation guidelines, children with cancer were offered online classmate “ambassador” visits during hospitalization.

**Objective:**

This study aimed to identify facilitators and barriers to online classmate “ambassador” visits during children with cancer’s hospitalization through a qualitative descriptive process evaluation using the Consolidated Framework for Implementation Research.

**Methods:**

From January to April 2022, we conducted 39 individual semistructured interviews with hospitalized children (n=16), their classmates (n=16), teachers from their schools (n=3), and study nurses (n=4) from involved hospitals. Most interviews (n=37, 95%) were conducted online using Microsoft Teams or Google Meet, while 2 (5%) interviews were conducted in person at the participants’ residences. This approach allowed us to gain a broad understanding of the facilitators and barriers to online ambassador visits.

**Results:**

We identified four themes: (1) working together, (2) ensuring participation, (3) staying connected, and (4) together online. The themes are described in terms of facilitators and barriers to online ambassador visits with 3 Consolidated Framework for Implementation Research domains: innovation, individuals, and the implementation process.

**Conclusions:**

Addressing the social needs of hospitalized children through online visits with their classmates may be relevant when one-on-one meetings are problematic. The online visits are highly dependent on collaboration between study nurses and teachers and assessing the needs of the hospitalized children. While a high degree of adult engagement and a stable internet connection are pivotal, these online visits can promote much-needed social interaction between children across physical settings.

## Introduction

In Denmark, 200 children aged between 0-18 years are diagnosed with cancer annually [1]. The positive advancement in treatment for childhood cancer has positively impacted the overall 5-year survival rates, which now exceeds an 80% chance for survival [2]. Although survival is expected for most children diagnosed with cancer, the treatment is often intense, involving chemotherapy, surgery, irradiation, and long periods of hospitalization [2,3]. As a result of the tough treatment, most children with cancer face long-term effects, which continue to plague them into their survivorship [3]. Childhood cancer survivors report long-term effects such as fatigue, impaired physical function, and poor cognitive function [4,5]. Furthermore, children with cancer and childhood cancer survivors also face social difficulties due to absenteeism from school, as well as leisure and social activities during treatment [6,7]. Consequently, their absence disrupts peer relationships, decreases social competencies, and increases feelings of loneliness [7,8]. Thus, treatment-related long-term effects combined with social isolation during treatment can hurt childhood cancer survivors’ long-term well-being [3,8].

When returning to school after treatment, childhood cancer survivors often experience uncertainty and fear of losing their social relationships with classmates or peers [9,10]. It is, therefore, essential that children with cancer stay connected with school during hospitalization to maintain a sense of normalcy, develop social skills, and ensure successful re-entry after treatment [11-13]. A 2016 systematic review shows that school re-entry programs and peer education of classmates about cancer can further promote positive attitudes toward the child with cancer [14]. This positive classmate attitude was associated with greater motivation for interacting with the child with cancer and including them in social situations [14]. In 2013, a multimodal intervention called RESPECT (Rehabilitation Including Social and Physical Activity and Education in Children and Teenagers With Cancer) was designed to target and ameliorate children with cancer’s physical, social, and academic functioning during hospitalization. The intervention included education of classmates about children with cancer and cancer treatment, visits by classmate “ambassadors” to the hospital, and establishing a link between the hospital and the child’s school peer group through in-hospital supervised activity [15,16]. Previous research from the RESPECT study has shown that social interaction by hospitalized children with cancer can promote a sense of connectedness with their classmates while, in turn, motivating the classmates to support them [17-19].

Recently, technologies, including video conferencing and telepresence robots, have become viable options for socialization and schooling [20,21]. These technologies have been shown to positively impact homebound or hospitalized children’s perceived social presence, academic behavior, and sense of normalcy [22-24]. As the COVID-19 pandemic shut down schools worldwide and the accompanying restrictions to physically isolate became inevitable, the need for technologies to support academic and social performance came to the forefront [25,26].

In response to the COVID-19 pandemic, hospitals and schools in Denmark prohibited in-person visits from March 2020 to April 2022, and the RESPECT study was forced to shift from in-person to online ambassador visits. We speculate that online visits may not be a better stand-alone option for school re-entry but could be seen as a supportive component. In some countries or settings, the online option may be more accessible than in-person visits, for example, a hospitalized child in isolation. However, it is still important to know the degree to which school re-entry programs and online ambassador visits complement each other and how online visits contribute. In this study, we aimed to identify facilitators and barriers to online ambassador visits for hospitalized children with cancer through a qualitative descriptive process evaluation using the Consolidated Framework for Implementation Research (CFIR). By identifying facilitators and barriers to online visits, this qualitative process evaluation intended to inform other health care professionals or professionals working closely with hospitalized children on what to be aware of when offering online visits to hospitalized children. Hopefully, our findings will inspire others to provide a social connection between hospitalized children and their peers.

## Methods

### Design

A qualitative descriptive process evaluation of online classmate “ambassador” visits was conducted with semistructured interviews after implementation. We used qualitative descriptive research to uncover the participants’ experiences through their descriptions of the online ambassador visits. Qualitative descriptive research design has been predominantly used within health care research to provide direct descriptions of phenomena where the experiences are described from the participants’ viewpoint [27]. In this study, qualitative descriptive research offers the opportunity to gather direct, rich descriptions of the online visits from the involved participants: hospitalized children, their ambassadors, their teachers, and the study nurses [27]. The knowledge gained from our participants’ descriptions can be used to design future online psychosocial interventions for hospitalized children. The CFIR was used to interpret the participants’ descriptions of facilitators and barriers to online visits [28]. As the meta-theoretical basis for the CFIR includes several implementation aspects, the CFIR provides a helpful framework for illuminating facilitators and barriers across 38 constructs within the five domains: (1) innovation, (2) outer setting, (3) inner setting, (4) individuals, and (5) implementation process [28]. The innovation domain refers to the proposed changes implemented and includes aspects of the intervention such as the innovation source and strength, evidence quality, relative advantage, adaptability, trialability, complexity, design, and cost [28]. The inner setting refers to the environment where the intervention is implemented, for example, hospital, school, city, etc. The inner setting domain includes team culture, compatibility, leadership engagement, and the implementation climate [28]. The outer setting domain refers to the context in which the intervention’s inner setting exists and includes patient needs and resources, the level at which the implementing organization is connected with other organizations, peer pressure, and external policies and incentives [28]. The individuals domain refers to personal beliefs, knowledge, self-efficacy*,* and attributes that affect the intervention’s implementation [28]. The implementation domain refers to activities and strategies used to implement the intervention, including planning, executing, reflecting, evaluating, and key intervention stakeholders, for example, opinion leaders, engagement, and project champions [28].

### The RESPECT Study

The initial RESPECT study was a controlled intervention study implemented at the University Hospital of Copenhagen from 2012 to 2019. An in-depth description of the RESPECT intervention study, including inclusion and exclusion criteria for participation, is described elsewhere [15,29]. Based on qualitative results from the RESPECT study [17,19], it was decided to implement this study nationwide, offering (1) educational sessions for classmates on cancer and treatment and (2) facilitation of classmate “ambassador” visits during hospitalization as an integral part of the RESPECT implementation study (Textbox 1).

**Overview of the RESPECT (Rehabilitation Including Social and Physical Activity and Education in Children and Teenagers With Cancer) intervention study and the RESPECT implementation study.**

**The RESPECT intervention study**. **Started in 2012. Ended in 2019.**

- Purpose: explore if involving healthy classmates at the hospital from the time of diagnosis and throughout treatment will improve the physical, educational, and social function of children with cancer, including facilitating their re-entry to everyday life after treatment.
- One intervention group from the pediatric oncology ward at the University Hospital of Copenhagen (Rigshospitalet).
- Three control groups from the pediatric oncology wards at Aarhus University Hospital, Aalborg University Hospital, and Odense University Hospital.

**The RESPECT intervention study consists of:**

- Educational sessions for classmates and teachers on childhood cancer, treatment, side effects, physical activity, and the RESPECT study in the school classroom by the study nurses.
- Supervised in-hospital physical activity. This component begins when the child with cancer or cancer-like disease is included in the RESPECT study.
- After the educational session, 2 classmates are elected as ambassadors in collaboration with the classmates, their parents, their teachers, and the study nurses.
- Classmate “Ambassador” visits during hospitalization. Ambassador visits are offered every 14th in-hospital stay day.

**June 2019**

- The national implementation of the RESPECT study began. The RESPECT intervention study changes to the RESPECT implementation study.

**The RESPECT implementation study. Started in 2019. Ongoing.**

- Purpose: to improve the social and educational well-being of children with cancer or cancer-like diseases during hospitalization, including facilitating their transition to everyday life after treatment.
- Offered at 4 Danish pediatric oncology wards: University Hospital of Copenhagen (Rigshospitalet), Aarhus University Hospital, Aalborg University Hospital, and Odense University Hospital.

**The RESPECT implementation study consists of:**

- Educational sessions for classmates and teachers on childhood cancer, treatment, side effects, physical activity, and the RESPECT study in the school classroom by the study nurses.
- After the educational session, 2 classmates are elected as ambassadors in collaboration with the classmates, their parents, their teachers, and the study nurses.
- Classmate “ambassador” visits during hospitalization. Online ambassador visits are offered every 14th in-hospital stay day.

**March 2020**

- COVID-19 pandemic hits Denmark, causing national lockdowns, including lockdowns at all 4 pediatric oncology wards and schools. No in-person ambassador visits are allowed. The RESPECT implementation study adapts to an online app format using Microsoft Teams (Microsoft) or Google Meet (Google).
- Online educational sessions for classmates and teachers on childhood cancer, treatment, side effects, and the RESPECT study by study nurses.
- Two classmates are chosen as ambassadors online in collaboration with the classmates, their parents, their teachers, and the study nurses.
- Online classmate “ambassador” visits during hospitalization. Online ambassador visits are offered every 14th in-hospital stay day.

### Participation in the RESPECT Implementation Study

Study nurses invited hospitalized children to participate in the RESPECT implementation study if they were (1) school-aged (6-18 years old); (2) diagnosed with cancer or cancer-like diseases, for example, immune deficiency or severe aplastic anemia; (3) treated with chemotherapy, radiation, surgery, or hematopoietic stem cell transplantation; and (4) receiving cancer treatment at a pediatric oncology ward in Denmark. Classmates were introduced to an “ambassador” function during the educational session in the classroom and could apply for the role, which involved visiting the hospitalized child throughout the treatment trajectory or until the classmate no longer wished to hold the function.

The classmates were also informed of the practicality of the ambassador visits, such as being transported to and from the hospital, that the time duration of the ambassador visits was always on a school day between 9 AM and 3 PM, and what to expect as an ambassador visiting the hospital, for example, seeing children who are sick. If needed, ambassadors were replaced with new ones. Further, 2 ambassadors per hospitalized child were identified in collaboration with the classroom teacher, the hospitalized child, the hospitalized child’s parents, the ambassadors’ parents, and this study’s nurses. All ambassadors were screened by this study’s nurses. The inclusion criteria for becoming an ambassador were (1) being a classmate to the hospitalized child and (2) possessing educational, emotional, and social competencies to support the hospitalized child. Children with cancer or cancer-like diseases and their classmates were excluded if they were (1) unable to speak Danish or (2) had severe mental disability.

### The RESPECT Implementation Study During the COVID-19 Pandemic

**Figure 1:**
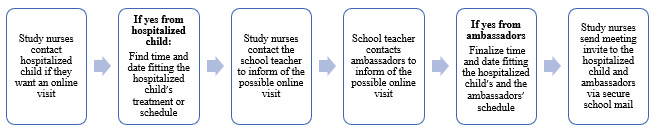
Preparation process for online ambassador visits.

To ensure that children with cancer stayed socially connected with their school classmates during the COVID-19 pandemic, the RESPECT implementation study adapted their in-person educational sessions and ambassador visits to a digital format using Microsoft Teams (Microsoft) or Google Meet (Google). Manuals or guidelines on how to organize or facilitate these online educational sessions or online ambassador visits were not developed before the initiation of the online visits. Guidelines were eventually developed simultaneously with conducting the online ambassador visits and based on experiences gained during the intervention. They were updated nationally during weekly meetings between this study’s nurses and the principal investigator of the RESPECT study. The online visits were led by study nurses associated with the RESPECT implementation study. The online visits were offered via secure mail accounts and were scheduled by this study’s nurses. These visits took place between 9 AM and 3 PM, with a frequency of 1 visit every 14th in-hospital day (Figure 1).

The online ambassador visits took place during regular school hours. The teachers were responsible for assisting the ambassadors with setting up their equipment, for example, logging in to Microsoft Teams or Google Meet but they were not required to be present during the online visits. The hospitalized children decided if they wanted their parents or this study’s nurse to be present during an online visit. In most cases, the parents did not participate but were present in the hospital room. The online visits did not have a fixed timeframe but ranged from 10 to 90 minutes, depending on the hospitalized children’s well-being on the day and the motivation of hospitalized children and their ambassadors to continue the session. Equipment such as computers, telephones, or tablets facilitated the online visits. The hospitalized children and the ambassadors often used computers provided by the school but a mobile device with an internet connection and secure mail account to access Microsoft Teams or Google Meet was acceptable.

### Participants and Recruitment

We recruited participants from the RESPECT implementation study for this study. All children with cancer or cancer-like diseases (hospitalized children), ambassadors, RESPECT study nurses, and teachers with experience in online ambassador visits were eligible to join this study. We used a convenience sample strategy to include the participants in this study. We strived to include participants representative from all of Denmark to provide nuanced descriptions of online visits. We included hospitalized children with cancer from 3 out of 4 hospitals that have a pediatric oncology ward. The criteria for inclusion of the hospitals were that the RESPECT implementation study must have been implemented and offered online visits during the COVID-19 pandemic.

**Figure 2:**
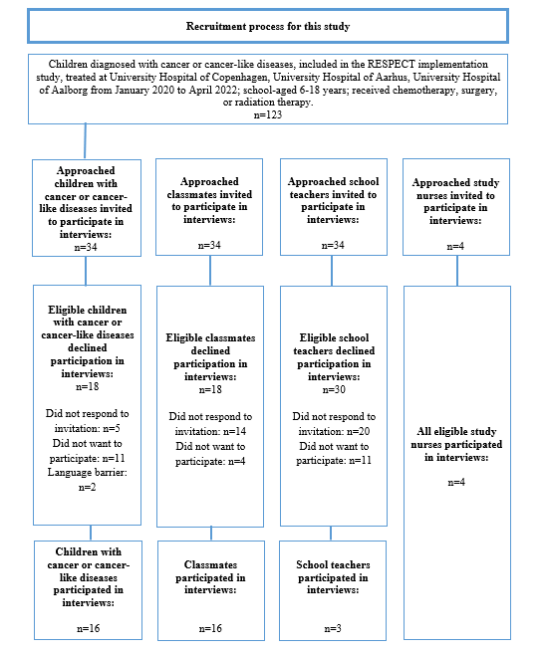
Recruitment process.

The implementation of the RESPECT implementation study was scheduled for the fourth hospital but was postponed due to the COVID-19 pandemic. As a result, the fourth hospital did not meet our inclusion criteria. From the RESPECT implementation study, 123 hospitalized children were identified as eligible to participate in this study. Out of these, this study’s nurses invited 34 hospitalized children to participate in this study. If the hospitalized child consented to participate, their ambassadors were also contacted regarding participation. Study nurses from the RESPECT study and schoolteachers were invited to participate if they had facilitated at least one online visit during the COVID-19 pandemic. Author NNB, a female PhD student without prior knowledge of the participants, contacted the invited participants by telephone regarding participation in this study. Of these 34 hospitalized children, 16 agreed to participate, as well as 16 of their ambassadors, 4 RESPECT study nurses, and 3 schoolteachers (Figure 2).

In total, 26 participants were from Zealand (University Hospital of Copenhagen), and 13 participants were from Jutland (University Hospital of Aarhus and University Hospital of Aalborg) in Denmark. The 16 hospitalized children (8 boys and 8 girls) were treated for leukemia (n=9), immune deficiency (n=3), extracranial solid tumors (n=2), tumors located in the central nervous system (n=1), or severe aplastic anemia (n=1). The hospitalized children and their ambassadors were aged between 7-16 (mean 10.5, SD 2.8) years. The hospitalized children participated in 4.5 (SD 3.5) online visits with a range of 1-12 online visits. Participant characteristics are presented in Table 1 below.

**Table 1:** Participant demographics. Note that ambassadors and schoolteachers are presented by region, not by hospital site, as they are not linked to the hospital.

Participant demographics				Children with cancer (n=16)		Ambassadors (n=16)		Study nurses (n=4)		Teachers (n=3)
**Sex, n (%)**										
	Male		8 (50)		6 (38)		—^a^		1 (33)	
	Female		8 (50)		10 (62)		4 (100)		2 (67)	
**Type of cancer or cancer-like diseases, n (%)**										
	Leukemia		9 (56)		—		—		—	
	Tumors located in the central nervous system		1 (6)		—		—		—	
	Extracranial solid tumors		2 (12)		—		—		—	
	Immune deficiency		3 (19)		—		—		—	
	Severe aplastic anemia		1 (6)		—		—		—	
**Treatment, (%)**										
	Chemotherapy		16 (100)		—		—		—	
	Radiation therapy		2 (12)		—		—		—	
	Surgery		4 (25)		—		—		—	
	Hematopoietic stem cell transplantation		4 (25)		—		—		—	
**Age (years),** **median (range)**										
	Age at the time of diagnosis		9.5 (6-14)		—		—		—	
	Age at the time of data collection		10.5 (7-16)		10.5 (7-16)		—		—	
**Region and pediatric oncology ward, n (%)**										
	**Zealand**		—		11 (69)		—		2 (67)	
		University Hospital of Copenhagen	11 (69)		—		2 (50)		—	
	**Jutland**		—		5 (31)		—		1 93)	
		Aarhus University Hospital	2 (12)		—		1 (25)		—	
		Aalborg University Hospital	3 (19)		—		1 (25)		—	

^a^Not available.

### Ethical Considerations

The RESPECT implementation study is a part of the original RESPECT study (file H-20077439). The Danish Data Protection Agency (file P-2021-208) approved the RESPECT study. Participants provided written and informed verbal consent, and parents gave consent on behalf of their children under 15 years of age. All participants were informed of their right to withdraw from this study at any time. Participants were pseudonymized for privacy considerations.

### Data Collection

In total, 4 semistructured interview guides were developed targeting the hospitalized children, ambassadors, teachers, and this study’s nurses (Multimedia Appendix 1). These guides were designed to elicit participants’ descriptions of online ambassador visits (including facilitators and barriers) and used open-ended questions to encourage reflection on their experiences [27]. The semistructured interviews were held from January to April 2022. Interviews were conducted by the first author (NNB), who introduced herself and this study’s aim before starting each interview. NNB has experience in performing qualitative interviews as well as in interviewing children across age groups. Some of the younger children (n=4) preferred being interviewed with their parents present for emotional support. No parent contributed to the interviews. Most interviews (37/39, 95%) were conducted using Microsoft Teams or Google Meet, while 1 hospitalized child and 1 ambassador preferred being interviewed at home. All interviews were audio recorded and varied in duration from 8 to 55 (mean 15.6, SD 10.7) minutes.

### Data Analysis

Interviews were transcribed verbatim by a student assistant using a transcription guide to ensure consistency. A Danish and English medical writer ensured that all descriptions were captured and interpreted correctly in English. A deductive analysis inspired by Braun and Clarke’s [30] approach to Thematic Analysis was used. The data were organized and coded using NVivo coding software. Due to our qualitative descriptive research design, we aimed to stay as close as possible to the participants’ descriptions of their experiences with online visits during the analysis process. CFIR was applied in the analysis as an interpretation tool to gain an in-depth understanding of facilitators and barriers to implementing online ambassador visits in a hospital setting. The analysis used the following steps: (1) the transcripts were read repeatedly until author NNB gained an in-depth understanding of the participants’ descriptions of the online classmate visits; (2) author NNB coded the participants’ descriptions into the CFIR domains; (3) authors NNB, MW, and HBL assigned the CFIR domains into overarching themes; (4) the themes were discussed and reviewed within the author group to ensure that the participant descriptions were rightfully captured within the CFIR domains and themes; and (5) themes were finalized after thorough discussion and agreement. All authors agreed on the final themes, and disagreements were discussed and resolved within the author group.

## Results

### Overview

We identified the following four themes from the participants’ descriptions of the online ambassador visits: (1) working together, (2) ensuring participation, (3) staying connected, and (4) together online. The themes included facilitators and barriers to online ambassador visits within 3 CFIR domains: innovation, individuals, and the implementation process.

### Working Together

Collaboration between the hospital and the school was an essential element when planning and facilitating online ambassador visits and included sharing information about the well-being and frame of mind of the children and technical support. Collaboration between the hospital and schools exemplifies the CFIR construct of “teaming” within the implementation process. This study’s nurses experienced collaboration with teachers as demanding when the teachers were not keen to secure online visits. These experiences were often linked to technical problems faced by the teachers or to their limited time.

Having the resources can be challenging for the teachers. They often say no thanks to the online visits because they do not have the resources. The teachers want to participate, but the practicality [of it] can be problem[atic]

Study nurse

Time constraints and technical issues led some teachers to disregard online visits. They were also seen as barriers that negatively impacted collaboration. The hospitalized children and their ambassadors never mentioned collaboration between the hospital and the school. Rather, the hospitalized children explained that they had a close relationship with this study’s nurses, which increased their willingness to participate in online visits. Collaboration between the hospitalized children and this study’s nurses was seen as a facilitator for ensuring participation in the online visits and also fit into the CFIR construct of “teaming” within the implementation process.

Most times, it is me who gets to decide what activities we do, but the nurse is the one who knows about my overall plan with school and my treatment during the day. I think it is nice [that] the nurse helps me because sometimes I don’t know what I want to talk about.

Hospitalized child aged 12 years

This study’s nurses elaborated that they depended on the hospitalized children’s collaboration to ensure that online visits occurred as intended, for example, ensuring social interaction. Collaboration between this study’s nurses and the hospitalized children exemplifies the CFIR construct of “planning” within the implementation process. When planning online visits, this study’s nurses identified their roles and responsibilities and defined the goals for success in collaboration with the hospitalized children.

### Ensuring Participation

Online ambassador visits required some level of adaptability to ensure the participation of the hospitalized children and their ambassadors. The teachers explained that schools used different online apps, such as Microsoft Teams and Google Meet, designed for emergency teaching during the COVID-19 pandemic. This study’s nurses adapted the setting of the online visits to fit each school’s preferred apps.

The CFIR construct “adaptability” within the innovation domain refers to how the intervention can be modified and tailored to fit into the inner settings [31]. In this study, the inner settings are the hospitals and schools where online visits are implemented. Using the schools’ preferred apps, this study’s nurses ensured that the teachers did not have to learn new technologies and had easy access to online visits. Thus, adapting to the setting was seen as a facilitator for online visits. This study’s nurses explained that they also adapted the content of online visits to ensure participation by the hospitalized children and their ambassadors.

…an unfocused, younger [or] sick child not feeling up to it or experiencing mood swings… that's when communication breaks down and [when] online visits become boring … that’s when I terminate the [online ambassador] visit.

Study nurse

Adaptability of the content of online visits also included changing the duration of the visit to accommodate the hospitalized child’s preferences and daily well-being. Adapting the content was described as a facilitator for the online visits. This study’s nurses’ decision regarding the duration of online visits was based on a “quality over quantity” value assessment. The children expressed that the timeframe for online visits was acceptable and that having a shorter timeframe did not impact their feelings of social connectedness.

This study’s nurses explained that they often participated actively during online visits if the hospitalized child desired their participation. The older children preferred to be alone with their ambassadors, whereas the younger children preferred the study’s nurses to take part in games or conversations.

I liked the privacy with my ambassadors during the [online] visits. I said to the nurse I wanted to be alone with my ambassadors so she started [Microsoft] Teams for me and then left. She [the nurse] came back when it was time to finish the visit.

Hospitalized adolescent aged 14 years

By assessing the needs of the intervention recipients, that is, the hospitalized children, this study’s nurses ensured that online visits met their needs, which CFIR underscores as important when implementing an intervention [31]. The younger hospitalized children experienced that this study’s nurses’ participation, including offering games or conversation topics, helped them feel socially connected to their ambassadors. This resulted in the hospitalized children being more interested in repeating the experience; hence, the participating role of this study’s nurse was seen as a facilitator of online visits.

…During the online visit, the two ambassadors started to play because they were physically together. I think they forgot [about] the hospitalized child. I tried to redirect their attention back to their ambassador visit, but I had to devise a game to keep them focused.

Study nurse

This study’s nurses described the younger children as having problems sitting still or staying focused for long periods of time. If a study nurse was unsuccessful in redirecting the children’s attention back to the visit, the visit would be terminated to ensure that any future online visits were perceived as positive experiences.

### Staying Connected

The hospitalized children explained that they were happy to see their ambassadors during online visits, as they often felt isolated from their school and social activities. The ambassadors also felt isolated from their school class due to the COVID-19 pandemic lockdown. As such, online visits provided an opportunity for both groups to sustain social interactions.

I got an insight into what was happening at school; how everyone was doing, and stuff like that. Knowing all of that helped me [during my hospitalization].

Hospitalized child aged 16 years

The hospitalized children described receiving information from their ambassadors, for example, about new classmates, new teachers, or the latest gossip, provided them with a sense of social connectedness.

Likewise, some ambassadors expressed how they experienced a sense of social connectedness with the hospitalized child, as the visits provided the ambassador with news about the hospitalized child’s life in the hospital. Understanding how the hospitalized children and their ambassadors experience connectedness during hospitalization is an example of the CFIR construct of “reflecting and evaluating” within the implementation process (ie, how successful the intervention is based on both qualitative and quantitative data) [31]. The ambassadors took great pride in their role and described it as being “information providers,” as such, the link between the hospital and the school, and as “supportive peers.” The most common task of the ambassadors was to share news from school.

She [the child with cancer] was happy that I told her [about what was going on in school]. She cheered up because I told her funny things like [the fact that] we got a new student in our class. She thought that was interesting.

Ambassador aged 11 years

This quote exemplifies how the ambassadors are happy to provide a sense of connectedness and social interaction as they perceive the hospitalized children’s feedback on sharing information from the school as positive. According to the CFIR construct “innovation delivers” within the implementation process domain, locating and understanding priorities from the innovation delivers is vital when implementing an intervention [31]. As innovation deliverers, the ambassadors’ motivation to participate in online visits stemmed from their desire to support the hospitalized child. The ambassadors also enjoyed receiving information about the everyday activities of the hospitalized children, reinforcing their feelings of social connectedness with the hospitalized children. However, the hospitalized children preferred receiving news and information from their ambassadors. Some of the teachers explained that choosing the right ambassadors to participate in online visits could enhance the social interaction experience.

Participating in online visits and socially interacting with each other can be difficult if they [the hospitalized child and their ambassadors] do not know each other. I think having [that] friendship before participating is important.

Teacher 2

If the hospitalized children and their ambassadors did not have preexisting relationships and had nothing in common to talk about, then social interaction would require more intervention by this study’s nurse. As such, ambassador selection can be seen as an important facilitator of social connectedness during hospitalization.

### Together Online

According to the construct “assessing context” within the implementation process, facilitators and barriers to implementing or delivering the intervention must be identified and assessed [31]. The hospitalized children and their ambassadors found the online ambassador visits favorable as they provided the opportunity to connect socially during hospitalization and the pandemic lockdown. However, both groups expressed that online visits could not replace in-person social interactions, including touching, playing physically together, or watching a movie together.

Being on Microsoft Teams is okay but not what I like. When you’re together in person, you can do a lot of things like run around.

Hospitalized child aged 9 years

Despite having no experience with in-person ambassador visits, the children still expressed that they preferred in-person visits to online ones and that online visits were more favorable than no visits at all. This study’s nurses described the online setting as a potential barrier to the children’s social interaction, as communicating face-to-face online was intense for most of the children, resulting in the children being shy and not knowing what to talk about. Likewise, the ambassadors expressed that being limited to talking was boring as they would have preferred to do physical activities together with the hospitalized child.

I would have liked to visit her [the hospitalized child] in the hospital. It was a bit boring online because all we did was talk. If we had been together in the hospital, we could walk around or sit together

Ambassador aged 11 years

Some of the hospitalized children mentioned that their treatment-related physical changes made them conscious of their appearance during the online visits, and the setting amplified their consciousness about their altered appearance. Consequently, the hospitalized children participated in online visits without using the camera. Their ambassadors stated that they did not feel as socially connected without being able to see the hospitalized child on the screen. The hospitalized children explained that being together with their ambassadors was more important to them than their altered appearance, so much so that, in some cases, they felt relaxed enough to participate online using the camera. The teachers also described the online setting as a potential barrier for ambassadors to understand the hospitalized children’s circumstances, including the severity of their disease or its treatment.

I think online visits can be difficult for the ambassadors because the setting is [similar to] watching a movie. Understanding the hospitalized child’s treatment through a movie [lens] can be difficult.

Teacher

Another potential barrier often seen in the setting of online visits is having a poor internet connection. This study’s nurses and teachers described how the hospital and school internet connections could fluctuate in terms of quality and negatively impact the integrity of the visits.

Sometimes, we canceled the online ambassador visits because of poor internet [connection]. That was a bit annoying because I had looked forward to seeing him [the hospitalized child]

Ambassador aged 13 years

Having a poor internet connection caused some ambassadors to feel discouraged that they could not keep their promise of social connectedness to the hospitalized children. Consequently, the hospitalized children and their ambassadors were not eager to participate in online visits when the internet was not fully functional.

## Discussion

### Overview

This qualitative process evaluation study aimed to identify facilitators and barriers to online ambassador visits during the hospitalization of children with cancer. Using qualitative descriptive research and CFIR, we gained an understanding of the online visit, including possibilities and difficulties faced by the hospitalized children, their ambassadors, schoolteachers, and study nurses. We found that the dominant facilitator was located within the construct of “teaming” in the implementation process domain, as the online visits required a high level of collaboration and adult facilitation. The main barrier was found within the “assessing context” construct in the implementation process domain, as the internet connection was considered a major barrier for online visits. Finally, further consideration should be given to the fact that, to date, online visits cannot provide the same level of social connectedness between children as physical visits.

In this study, close collaboration between this study’s nurses and the teachers and close collaboration between this study’s nurses and hospitalized children were seen as pivotal facilitators of online visits. However, strategies regarding implementation and collaboration are needed to specify the involvement of the various players associated with the intervention. CFIR suggests careful consideration of the individual’s capability (interpersonal competence, knowledge, and skills to fulfill their role) and their motivation for fulfilling their role when implementing an intervention [31]. Based on our findings, we suggest that assessing the individuals’ capability can potentially strengthen collaboration, as we found that identifying and outlining the roles of the individuals delivering the intervention and collaboration between this study’s nurses and the hospitalized children formed the basis for successful integration of the online visits and led to strengthening feelings of social connectedness. Other studies suggest involving stakeholders, for example, health care professionals, teachers, and children, is vital to successful implementation [32-34]. However, based on our findings, strategies or guidelines on how to ensure collaboration when involving different stakeholders such as study nurses, teachers, hospitalized children, and ambassadors should be considered as we found that the collaboration between this study’s nurses and teachers was not without challenges.

Another finding was that the hospitalized children had individual needs and preferences for the content of the online visits and the study’s nurses’ participation. Likewise, previous research suggests that pediatric interventions are not one-size-fits-all, and to ensure participation and involvement, there must be an element of individualization, that is, accommodating an individual’s needs and preferences [35-37]. Our findings suggest that, depending on age, children interacted differently. Young-aged children were especially impacted, which was considered a potential barrier to online visits. Younger children needed more facilitation, and as such, this required more time commitment from this study’s nurses. Interestingly, a study from 2013 reported similar findings, which included that time commitments were a barrier to their nurse-led videoconferencing intervention [38]. Considering that our study finding is similar to the study from 10 years ago, further research on how to accommodate time commitment issues should be actively pursued. We argue that setting achievable goals for each online visit should be required and that these goals should be discussed with all participating children. Regardless, research on actively involving intervention recipients in designing and adapting interventions for children is needed. When offering pediatric interventions, the adaptability of the intervention also requires consideration. In our study, both the setting and the content of the online visits were adaptable. This ensured participation and supported a feeling of being socially connected during the COVID-19 pandemic.

This study shows that online visits can lead to social connectedness between hospitalized children and their ambassadors. A central finding is that receiving new information during the visits supported the hospitalized children’s feelings of social connectedness. Similarly, Pennant et al [39] found that hospitalized children and young adults benefited from social support during treatment, as social support was associated with feelings of “not being alone” and better coping strategies [40]. Our findings suggest that knowledge about each other and the children’s pre-existing relationships facilitated social interaction. Therefore, exploring the children’s motivation for participating in online visits could enhance feelings of social connectedness, limit the need for adult supervision, and thus reduce time consumption by this study’s nurses.

Lastly, it is important to note that this study’s nurses and teachers considered the online setting a barrier to social interaction. In contrast, the children proclaimed that the online setting presented an opportunity to interact socially and gave them a sense of connectedness. These findings align with previous research showing that videoconferencing technologies can strengthen friendships and relationships with peers and provide a sense of social presence for children with chronic diseases [24,38]. Although the online setting provided an opportunity to interact socially, our findings showed that the children preferred in-person visits to online visits as the online visits were perceived as boring in some situations. However, the online visits were better than no visits at all. Therefore, we suggest online visits may be a good option for hospitalized children in the absence of an in-person option but that in-person visits remain preferable. Furthermore, our findings show that a good internet connection is indispensable when offering online visits as a poor internet connection can impact the children’s motivation to participate and ultimately lead to negative views about social interaction online. Likewise, Weibel et al [24] found that poor internet connection and audio capabilities limited the children’s use of telepresence robots. Johannessen et al [41] also argued that having a poor internet connection is problematic for online dialogue. This is supported by previous research showing that poor internet quality is the primary source of technological difficulties when using online formats [42-44]. Although these studies report that poor internet connection is problematic for online social interaction or web-based interventions, none of the studies provide any solutions.

### Limitations

Most (9/16, 56%) of the hospitalized children involved in this study were diagnosed with leukemia, and the remaining 7 (44%) children were diagnosed with other cancers or cancer-like diseases. Thus, further work is needed to explore whether this study’s findings are transferable to children with other diagnoses. Furthermore, only 3 (9%) of the 34 eligible teachers wanted to participate in this study, which limited variety in the description of online ambassador visits.

A central limitation of this study was that CFIR was not formally applied when developing the interview guides but only during data analysis. Consequently, we did not address constructs from the outer setting and inner setting domains. Hence, some key elements require further consideration. The online ambassador visits were part of an existing study offering in-person ambassador visits during hospitalization. This study has been ongoing since 2013, with several guidelines for in-person ambassador visits. Furthermore, this study’s nurses connected to this study were familiar with organizing and facilitating in-person ambassador visits, which may have impacted their readiness for change when the COVID-19 pandemic resulted in physical isolation across Denmark. Another example is how schools in Denmark changed from an in-person classroom teaching format to an online context during the COVID-19 pandemic. This impacted how the participants adapted to the online ambassador visits [45]. The schools’ readiness for change may also have impacted how the participants adapted to the online ambassador visits. Previous results from the RESPECT study show that children with cancer and their classmates are motivated to participate in in-person classmate visits [17,18,29]. Thus, we believe that the combination of an already existing organizational structure derived from our experiences with in-person ambassador visits and our knowledge of the classmates’ motivation to participate in in-person ambassador visits has enhanced this study’s nurses’ flexibility to adapt from the in-person to the online context. Therefore, we are unsure if implementing online ambassador visits in other settings may differ from ours.

### Future Perspective

Emerging research highlights the importance of staying socially connected with peers and classmates during cancer treatment [37,39,46] and that there is a need for interventions that specifically target the relationship between children with cancer and their classmates [11,47,48]. Our study findings suggest that online visits with classmates or ambassadors can meet the hospitalized child’s need for social connectedness, albeit in-person visits are preferable for some children. Accordingly, online ambassador visits may prove valuable when in-person visits are not an option due to long distances and hospital isolation. However, the same finding also warns of the need to be aware of the children’s ages and individual preferences. Thus, it is advisable to assess and address the needs of the participating children, including the differences in needs across ages and individual preferences. While poor internet connection can negatively impact online social interaction, knowledge about how to accommodate internet consistency is limited. Future web-based interventions should consider establishing an appropriate internet connection to ensure participation. Based on our findings, we suggest that health care professionals offering online visits to hospitalized children align their expectations with those of the participating children regarding the purpose and context of the visits. This study’s findings may be transferable to other pediatric settings as knowledge about online visits and their facilitation during hospitalization can be applied across disease groups and cultures. However, research is needed to understand how to implement online visits in other contexts.

### Conclusions

Hospitalized children and their ambassadors benefited from participating in the online ambassador visits as these visits contributed to enhanced social connectedness. This study’s findings showed that online social interaction between hospitalized children and their ambassadors is possible but requires being attentive to the individual needs of the hospitalized children and continuous collaboration between the hospital and school regarding organizing and facilitating. The online visits were pivotally reliant on sound internet connections. Including classmates during treatment should not be underestimated when addressing the social needs of hospitalized children. Future online psychosocial interventions can advantageously consider the collaboration between involved participations, sensitivity regarding individual preferences, and creating a stable internet connection when offering online visits during hospitalization.

## Appendix

Multimedia Appendix 1 Interview guides for hospitalized children, classmates (ambassadors), study nurses, and teachers.

## Abbreviations

CFIR: Consolidated Framework for Implementation Research
RESPECT: Rehabilitation Including Social and Physical Activity and Education in Children and Teenagers With Cancer
