# Supplementary material for: Online Ambassador Visits for Hospitalized Children With Cancer: Qualitative Evaluation of Implementation
Source: JMIR Pediatr Parent. 2024 Sep 4;7:e53309. doi: 10.2196/53309 (PMC11411222; doi:10.2196/53309)
Supplement: Multimedia Appendix 1 [file pediatrics_v7i1e53309_app1.docx]

# Interview guide for the hospitalised children

## Introduction to the interview

Today, we will talk about your experiences with being online with two of your classmates during your hospitalization. I am very interested in your general opinion about these online classmate interactions, such as what has been good or bad, and your opinion about being online with your classmates.

## Information about the child with cancer

Q1: Can you tell me a bit about yourself? How old are you, and what school level are you in?

Q2: What do you like to do in your spare time?

Q3: How long have you been sick?

Q4: Are you in school right now (physical, online, or not at all)?

## The online classmate interaction

Q5: Who are your classmates that visit you online?

Q6: Can you describe a typical day when you are together with your classmates online?

Q7: Can you tell me a bit about your classmates when you meet online?

- What do you do – do you play any games?
- What do you talk about – school, peers?
- How long is the online social interaction?
- Do you think you got enough time with your classmates (or would you have preferred to have a shorter/longer time)?
- How often do you meet with your classmates?
- Do you that that is enough? Why/why not?

Q8: What do you like about online social interactions?

Q9: What do you not like about them?

Q10: How was meeting your classmates online for the first time?

- Can you tell me what you did?

Q11: Is it different being online with your classmates than if you had been together physically?

- How/how not?

Q12: What do you think is essential to know for other children with cancer if they had to be online with their classmates?

- Do you have any good ideas as to what they can do to get a good visit?
- If other children with cancer did not know if they wanted to be together with their classmates online, what would you tell them?
- Would you tell them any bad things related to online classmate visits?

## Help from study nurses and teachers

Q13: How did the study nurses or teachers help you with the online classmate interactions?

- Can you describe how they helped you – did they plan any activities?

Q14: Does the study nurse or your teacher participate in the online interactions with your classmates?

- Can you describe what the study nurse or the teacher does during the online interactions?
- What do you think about these activities? Do you like them, dislike them, or are they unneeded?

## At the end of the interview

That was all I had for today. Is there anything else you want to tell me before I stop the Dictaphone? Thank you so much for participating in this interview.

# Interview guide for ambassadors

## Introduction to the interview

Today, we will talk about your experiences with being online with your classmate in the hospital. I am very interested in your general opinion about these online classmate interactions, such as what has been good or bad, and your opinion about being online with your classmate.

## Information about the child with cancer

Q1: Can you tell me a bit about yourself? How old are you, and what school level are you in?

Q2: What do you like to do in your spare time?

## The online classmate interaction

Q3: Who is your classmate in the hospital?

Q4: Can you describe a typical day when you are with your classmate online?

Q5: Can you tell me a bit about your classmate when you meet online?

- What do you do – do you play any games?
- What do you talk about – school, peers?
- How long is the online social interaction?
- Do you think you got enough time with your classmate (or would you have preferred to have a shorter/longer time)?
- How often do you meet with your classmate?
- Do you that that is enough? Why/why not?

Q6: What do you like about online social interactions?

Q7: What do you not like about them?

Q8: How was meeting your classmate online for the first time?

- Can you tell me what you did?

Q9: Is it different being online with your classmate than if you had been together physically?

- How/how not?

Q10: What do you think is essential to know for other classmates if they had to be online with their classmates in the hospital?

- Do you have any good ideas as to what they can do to get a good visit?
- If other children with cancer did not know if they wanted to be together with their classmates online, what would you tell them?
- Would you tell them any bad things related to online classmate visits?

## Help from study nurses and teachers

Q11: How did the study nurses or teachers help you with the online classmate interactions?

- Can you describe how they helped you – did they plan any activities?

Q12: Does the study nurse or your teacher participate in the online interactions with your classmate?

- Can you describe what the study nurse or the teacher does during the online interactions?
- What do you think about these activities? Do you like them, dislike them, or are they unneeded?

## At the end of the interview

That was all I had for today. Is there anything else you want to tell me before I stop the Dictaphone? Thank you so much for participating in this interview.

# Interview guide with teachers and Study nurses

## Introduction to the interview

Today, we will talk about your experiences with online classmate interactions. I am very interested in your general opinion about these online classmate interactions, such as what has been good or bad, your idea about facilitating them, and if you experienced any difficulties with the online classmate interviews.

## Introduction to the participant

Q1: What is your occupation, and what are your work assignments?

Organizing the online classmate interactions

Q2: Can you describe the typical online classmate interaction?

- How long is a typical online interaction?

Q3: How do you organize online classmate interactions?

- What challenges do you meet when organizing online classmate interactions?

Q4: What is your experience of online interactions?

- What do the children with cancer tell you about online interactions?
- What do the classmates tell you about online interactions?

Q5: Can you describe a good online interaction?

- Do you do anything to make the online interaction good – how?

Q6: Can you describe a bad or less good online interaction?

- Such as a specific situation?

## Facilitating the online classmate interaction

Q7: How were you introduced to online interactions?

- Do you remember any guidelines which you had to follow?

Q8: Who creates the framework for online interactions?

- Do you experience any online interactions that require more facilitation than others?
- Why do you think that?

Q9: What kind of thoughts do you have regarding the social and educational framework for online interactions?

Q10: How do you plan the content of the online interactions?

- Do you involve the children with cancer or the classmates in the planning?
- How do you involve the children with cancer or their classmates?
- Do you experience that involving children with cancer or classmates has a positive/negative impact on online interactions?
- Are there any activities that don’t make any sense to plan?
- Why?

Q11: What kind of considerations do you have regarding strengthening the social relation between children with cancer and their classmates?

Q12: Have you experienced that some children need to tell you about online interactions afterward?

- If yes: how did you handle this?

Experiences and good advice

Q13: Do you experience online interactions making sense for children with cancer or their classmates?

- If yes: are there any situations where an online interaction did not make sense?

Q14: What do you think is essential to know for others planning on facilitating an online interaction between children?

- For some who don’t have any experience with it?

Q16: Can you name some of your experiences that you would pass on to the next person?

## At the end of the interview

This completes the interview. Is there anything else you think would be vital for me to know? Thank you so much for participating.
